# Evaluating the reliability of YouTube videos as an educational source on heart failure

**DOI:** 10.1590/1806-9282.20250438

**Published:** 2026-05-01

**Authors:** Serkan Bulguroglu, Mehmet Erdogan

**Affiliations:** 1 University of Health Sciences, Hatay Dörtyol State Hospital, Department of Cardiology – Hatay, Turkey.; 2 Ankara Yıldırım Beyazıt University, Medical School, Department of Cardiology – Ankara, Turkey.

**Keywords:** Heart failure, Social media, Health education, Health communication

## Abstract

**BACKGROUND:**

YouTube, a video-sharing platform, aids health information sharing, and while social media’s role in heart failure care remains unclear, it can enhance interaction, education, and engagement, fostering patient-centered care and encouraging treatment adherence and active health management.

**OBJECTIVE:**

The aim of the study was to evaluate the quality and usability of heart failure-related YouTube videos as a source of information for patients.

**METHODS:**

A total of 100 English-language YouTube videos on heart failure were analyzed. Videos were categorized based on uploader identity (healthcare vs. non-healthcare professionals) and assessed using quality criteria for consumer health information, Global Quality Scale, Journal of the American Medical Association criteria, and Video Power Index. Quantile regression analysis was performed to identify independent predictors of video quality.

**RESULTS:**

Of the videos analyzed, 69% were uploaded by healthcare professionals. The mean quality criteria for consumer health information score was 21, Global Quality Scale was 3, and Journal of the American Medical Association was 3. Videos from professionals and longer videos had significantly higher quality scores. Quantile regression showed that video duration predicted high Global Quality Scale values at the 75th and 90th percentiles, while professional source was a consistent predictor across most quantiles.

**CONCLUSION:**

The overall quality of YouTube videos on heart failure was found to be low to moderate, with substantial room for improvement. Videos uploaded by healthcare professionals, however, consistently demonstrated higher quality across evaluation metrics. Longer videos tend to have higher quality, but popularity does not correlate with content reliability. Efforts should be made to improve video content for better patient education.

## INTRODUCTION

Heart failure is an important disease that causes increased health expenditures and decreased quality of life^1^. The chronic and progressive disease poses a significant burden to patients^1^. The number of patients with heart failure in our country in 2021 was determined to be over one million, and the cost to the health system has reached one billion dollars^2^. The impact of heart failure on healthcare systems is profound, as the disease’s trajectory involves frequent flare-ups necessitating repeated and extended hospital stays, particularly in the final 2 years of life^3^. The shortage of medical resources complicates efforts to address the consultation and treatment needs of heart failure patients^4^.

According to recent data from the Pew Research Center, a rise in seeking health-related content on YouTube has been documented, with approximately 59% of US adults reporting watching health-related videos in the past year. These numbers underscore the platform’s critical role in disseminating health information^5^. YouTube is an internet video-sharing platform that increasingly serves as a tool for searching and disseminating health information^6^. But social media use in heart failure care is still not fully understood. Social media could improve interaction while encouraging education and involvement in heart failure for both healthcare providers and patients. This can facilitate evidence-based, patient-focused care by healthcare providers and inspire patients to adhere to treatments and actively manage their own health^5^. In this study, we aimed to assess the quality levels of videos related to heart failure and evaluate their usability by patients for informational purposes.

## METHODS

We conducted a search for YouTube videos on heart failure using the keyword “heart failure” on July 1, 2024, using a new profile to avoid search bias. YouTube offers four sorting options: relevance, upload rate, view count, and rating. We selected 100 videos based on relevance^7^, excluding unrelated, ad-containing, repetitive, or silent videos. The “relevance” option was intentionally selected because it approximates how an average YouTube user would encounter results for the keyword “heart failure.” To minimize algorithm-driven variability, the search was conducted in a newly created, unsigned-in browser session with cache, cookies, and watch history cleared, and with the location fixed to a single region. Other filters such as “view count” or “rating” were not preferred, since they disproportionately prioritize older or entertainment-focused videos rather than those most visible to new information seekers. This strategy has been used in several peer-reviewed video-analysis studies and improves ecological validity. A total of 100 videos were analyzed, which aligns with prior YouTube content-quality investigations (typically 50–150 videos) and provided sufficient thematic coverage while remaining feasible for dual expert review^8,9^. Data collected included views, comments, likes, dislikes, length, upload time, and channel subscribers. Video uploaders were categorized as health or non-health professionals. Because the study focused on the experience of a typical information-seeking YouTube viewer, videos were evaluated from a general-audience perspective without formal classification by intended audience. YouTube does not routinely provide reliable metadata indicating whether videos target professionals or patients, making systematic categorization impractical. Ethical approval was not required because all analyzed materials were publicly available. No personal identifiers were used, and video creators were anonymized in the dataset. The study adheres to the principles of the Declaration of Helsinki regarding the use of publicly accessible data. This manuscript followed STROBE (Strengthening the Reporting of Observational Studies in Epidemiology) guidelines for cross-sectional studies. Videos were evaluated using established scoring systems. The Global Quality Scale (GQS) assessed quality from 1 (poor) to 5 (excellent)^10^. Videos scoring 4–5 were considered good quality, 3 medium, and 1–2 poor. The DISCERN (Quality Criteria for Consumer Health Information) tool evaluated content reliability, assessing clarity, relevance, balance, and treatment information accuracy^11^. Based on scores, videos were classified as excellent (63–75), good (51–62), fair (39–50), weak (27–38), and very weak (<27)^8^. The JAMA (Journal of the American Medical Association) benchmark criteria assessed source trustworthiness, scoring 0–4^9^. This methodology aligns with prior video content analyses by Goobie et al., He et al., and Singh et al.^8,9,10^. The Video Power Index (VPI) was calculated to evaluate both the popularity and audience engagement level of each video. To evaluate the popularity of the videos, average daily views [total views/days since upload], VPI {[like × 100/(like+dislike)] × (views/day)/100}, and like ratio [(likes/likes+dislikes) × 100] were calculated for each video. Two cardiologists (15 and 8 years’ experience) evaluated videos following European Society of Cardiology and American Heart Association guidelines^12^. Inter-rater reliability showed substantial agreement: GQS Cohen’s kappa=0.79, DISCERN=0.78, JAMA=0.84. Rare disagreements (n=2) were resolved through a third senior reviewer.

### Statistical analyses

The analyses were performed using IBM SPSS Statistics for Macintosh, Version 29.0 (IBM Corp., Armonk, New York, USA). Data dispersion was tested using the one-sample Kolmogorov-Smirnov test. For non-normally distributed continuous data, the Kruskal-Wallis test was applied, with results as median with interquartile range. The Bonferroni correction test was used for pairwise comparisons. Chi-square tests were applied for categorical variables and Fisher’s Exact test when necessary. Results were presented as n (%). Spearman correlation analysis assessed relationships between video quality scores and baseline features. Correlation analyses were conducted in an exploratory and descriptive manner to summarize relationships among variables and to identify candidate predictors for the subsequent quantile regression model. As these correlations were not used for inferential hypothesis testing, no formal multiple-comparison correction was applied. The GQS, our primary outcome variable, showed a skewed distribution with non-constant variance and non-linear predictor relationships, violating standard linear regression assumptions. Quantile regression was selected as a robust alternative to estimate conditional quantiles of the dependent variable, allowing investigation of predictor effects across GQS levels. Because GQS scores were non-normally distributed and displayed heteroscedastic residuals, we employed quantile regression to model conditional quantiles (0.25, 0.50, 0.75, and 0.90) rather than mean values. This technique estimates how predictor effects vary across the distribution of video quality, allowing assessment of whether factors such as video duration or professional source influence lower-versus higher-quality strata. Confidence intervals were generated using standard errors, and the Wald test calculated p-values. A twosided p-value <0.05 was considered significant.

## RESULTS

Data was collected from 100 videos after applying the inclusion and exclusion criteria. The videos were classified into two categories based on uploaders: medical professionals (69%) and non-medical users (31%). The average upload time was 5 years (2–9), with a median duration of 526 s (298–1,106). Videos had median likes of 2,800 (1,800–4,700), dislikes of 49 (24–93), and 131 comments (64–245). The median views were 1,63,000 (1,00,000–3,41,000), and subscribers were 6,99,000 (1,65,000–17,17,000). The average DISCERN scale score was 21 (16–37), the JAMA score was 3 (2–3), GQS was 3 (2–3), and the video power index was 97.9 (97.2–98.9) (Table 1). Table 1 shows relationships between video quality scores and features. A significant positive correlation existed between video duration and source across all scoring systems, while other parameters showed no significant relationships. Video characteristics and GQS comparison showed that longer duration and medical professional sources corresponded to higher quality scores (Table 2). Quantile regression analysis of GQS revealed that video duration was significantly above the 50th quantile, especially in the 75th and 90th quantiles, while healthcare professional sources predicted GQS across all quantiles except the 75th (Table 3).

**Table 1:** The relationship between video quality scores and video features.

Variables	Global quality score		DISCERN quality score		JAMA score	
	r coefficient	p-value	r coefficient	p-value	r coefficient	p-value
Duration of video	0.42	<0.001	0.42	<0.001	0.30	<0.001
Number of views	0.02	0.852	-0.05	0.653	-0.09	0.363
Number of likes	0.10	0.344	0.10	0.31	0.01	0.918
Number of dislikes	0.10	0.351	0.06	0.583	0.03	0.803
Video power index	0.17	0.111	0.16	0.131	0.01	0.911
Number of comments	-0.02	0.835	0.03	0.773	0.08	0.407
Number of subscribers	0.24	0.018	0.25	0.012	0.16	0.11
Duration since upload	-0.17	0.084	-0.22	0.03	-0.15	0.145
Video source (Ref: health professional)	0.4	<0.001	0.36	<0.001	0.52	<0.001

JAMA score: Journal of the American Medical Association benchmark criteria; DISCERN: quality criteria for consumer health information. Data were presented as the r coefficient (Spearman’s rho) and p-value.

**Table 2:** Comparison of video characteristics and quality scores for video content quality.

Variables	Low GQS (Score 1–2) (n=43)	Intermediate GQS (Score 3) (n=34)	High GQS (Score 4–5) (n=23)	p-value
Duration of video (seconds)	366 (153–792)	529 (355–1,001)	1,119 (636–3,138)	<0.001
Number of views (×10^3^)	130 (92–341)	219 (126–358)	126 (93–287)	0.284
Number of likes (×10^2^)	25 (10–41)	32 (21–57)	25 (17–47)	0.320
Number of dislikes	43 (19–89)	31 (59–100)	49 (27–87)	0.283
View ratio (×10^3^)	41.8 (16.8–81.7)	51.0 (21.1–123.3)	57.0 (24.2–180.0)	0.138
Like ratio	97.9 (97.2–98.7)	97.8 (97.3–98.8)	98.4 (97.0–99.1)	0.946
Video power index (×10^3^)	41.3 (16.4–84.7)	62.3 (20.9–130.4)	56.1 (23.9–175.5)	0.160
Years since upload	5.0 (3.0–9.0)	4.5 (2.0–8.2)	4.0 (2.0–7.0)	0.322
Number of comments	158 (54–286)	117 (59–259)	124 (73–178)	0.855
Number of subscribers (×10^3^)	316 (560–1,150)	770 (229–1,770)	755 (472–2,150)	0.060
Video source				0.002
Medical professionals	22 (51%)	26 (76%)	21 (91%)	
Non-medical users	21 (49%)	8 (24%)	2 (9%)	
Global quality score	2.0 (1.0–2.0)	3.0 (3.0–3.0)	4.0 (4.0–5.0)	<0.001
DISCERN Quality score	16.0 (9.0–24.0)	21.0 (20.0–35.0)	43.0 (26.0–51.0)	<0.001
JAMA score	2.0 (1.0–3.0)	3.0 (2.0–3.0)	3.0 (3.0–3.0)	0.002

GQS: global quality score. JAMA score: Journal of the American Medical Association benchmark criteria; DISCERN: quality criteria for consumer health information. Data were presented as median (25th–75th percentile) and n (%).

**Table 3:** Multivariable quantile regression analyses predicting the Global quality score.

Variables	10th quantile Coefficient (95%CI)	25th quantile Coefficient (95%CI)	50th quantile (median) Coefficient (95%CI)	75th quantile Coefficient (95%CI)	90th quantile Coefficient (95%CI)
Intercept	1.07 (0.26–1.87)^*^	0.99 (0.35–1.64)^*^	2.00 (1.08–2.92)^**^	2.68 (1.99–3.37)^**^	2.86 (2.06–3.66)^**^
Video duration (per sec)	0.01 (0.00–0.01)	0.00 (0.00–0.01)	0.00 (0.00–0.01)	0.01 (0.00–0.01)^**^	0.01 (0.00–0.01)^**^
Video source (health professional)	0.99 (0.27–1.72)^*^	0.98 (0.40–1.56)^*^	0.97 (0.14–1.80)^*^	0.23 (-0.39–0.85)	0.95 (0.23–1.68)^*^
Time since upload (years)	-0.01 (-0.10–0.09)	-0.01 (-0.07–0.07)	-0.01 (-0.11–0.10)	0.01 (-0.10–0.08)	0.01 (-0.09–0.09)

Notes: *p<0.05;

**p<0.001 (based on Wald test). Values represent regression coefficients and 95%CI from quantile regression models. Dependent variable: Global Quality Score (GQS). Independent predictors: video duration (continuous), video source (binary), and upload age. CI: confidence interval.

## DISCUSSION

The top 100 YouTube videos related to heart failure were evaluated by two experienced cardiologists using quality scoring systems: GQS, JAMA, DISCERN, and VPI. Our findings indicate heart failure-related videos on YouTube are generally of low quality, with room for improvement in source transparency, balance of information, and evidence-based content, though longer videos had higher quality scores. Videos uploaded by healthcare professionals scored higher than those by non-professionals. No correlation was found between video quality scores and popularity factors like views, likes, comments, or subscribers. While no correlation existed between popularity metrics and video quality scores, this isn’t necessarily negative. YouTube’s algorithms often prioritize viewer engagement over educational value. Videos with sensationalized thumbnails or emotionally charged content may be promoted more frequently, regardless of accuracy. High-quality videos by healthcare professionals may receive less visibility despite scientific rigor. This platform-related bias can hinder reliable health information dissemination. Video duration was positively associated with GQS only in the upper quantiles (75th and 90th percentiles), indicating that longer videos contributed meaningfully to quality only among higher-performing content, whereas healthcare- professional uploads had consistent effects across quantiles. Previous studies have assessed YouTube health content using various quality indices; however, few have examined cardiovascular topics through the lens of quantile regression modeling. This approach allows for a more granular understanding of how factors such as video source, duration, and audience type influence quality across the distribution of content reliability. The scoring systems used have been employed in similar studies, proving suitable for assessment^8,9,11,12,13,14^. The JAMA, DISCERN, and GQS systems are commonly used to evaluate video quality, and YouTube has implemented certification guidelines for content reliability. Although healthcare professionals were expected to upload higher-quality videos, only 69% were from healthcare professionals. Their videos were generally more reliable, based on current guidelines and scientific literature. While healthcare professionals’ videos were anticipated to have higher quality, their content wasn’t always as high as expected. However, consistent with studies on other medical topics, healthcare professionals’ videos scored better on all quality metrics than non-professionals^8,9,11^. The use of guidelines and scientific accuracy contributed to these videos’ higher quality. Therefore, videos from healthcare professionals should be prioritized for accurate heart failure information.

This study did not systematically assess the intended audience of the analyzed videos, though some may have been designed for healthcare professionals or medical students. While such videos may score highly in quality, they might be difficult for laypersons to understand. Future studies should consider classifying videos based on intended audience to better evaluate content quality and communication effectiveness.

We prefer quantile regression due to clear violations of linear regression assumptions in our dataset. The skewed GQS distribution and heteroscedasticity suggested mean-based models could lead to biased inferences. Quantile regression enabled detection of differential effects across quality tiers, offering insights masked in average-based analyses.

Video duration increased quality scores, as longer videos share more detailed information. However, quantile regression showed the source was more significant than duration, indicating uploader expertise is crucial for quality. Unlike other studies, we found no correlation between quality scores and video popularity.

The findings highlight the urgent need for collaboration between medical institutions and digital platforms to improve online health information. YouTube could integrate content verification labels, prioritize videos reviewed by certified health organizations, and refine algorithms to reduce the visibility of misleading videos. Additionally, enhancing digital health literacy among the public would empower users to critically evaluate online medical content.

Patient education is crucial in managing heart failure, helping improve self-care and treatment adherence^14,15^. However, some videos contained misleading information about “miraculous” treatments lacking scientific evidence. Since heart failure patients often seek online guidance, video-based education is important. While YouTube plays a significant role, many videos were inadequate.

Healthcare professionals’ involvement is essential for ensuring content accuracy. YouTube should enhance oversight using algorithms to detect misleading videos. A healthcare committee could help monitor content. Our study indicates a need for comprehensive videos aligned with medical guidelines. YouTube should encourage series videos and improve user access to enhance patient knowledge and treatment adherence.

This study provides important insights for healthcare professionals to guide patients to reliable video sources and encourages patients to critically evaluate video credibility. With the increasing use of YouTube for health-related information, it’s crucial to develop algorithms and regulatory oversight to ensure the accuracy of shared content.

### Limitations of the study

This study has several limitations. First, only English-language videos were analyzed, and other linguistic or cultural contexts were not explored. Production quality, visual aids, and presenter credentials were not systematically evaluated. The study did not assess adherence to contemporary clinical guidelines such as the 2023 ESC (European Society of Cardiology) recommendations for heart failure management. Given the cross-sectional design, the dynamic and continuously changing nature of YouTube content could not be captured. YouTube’s “relevance” ranking is algorithm-based and dynamic; video ordering may vary by time or region. Although we standardized search conditions, this limitation may affect reproducibility. Although some videos were clearly produced for healthcare professionals or medical students, the target audience could not be objectively verified from available data. Consequently, professionally oriented content may have contributed to higher quality scores despite being less comprehensible to patients. Future investigations should include explicit audience stratification to determine how accessibility and scientific meticulousness interact. Finally, potential confounding variables, including audience type and engagement bias, may have influenced the observed relationships.

## CONCLUSION

In this study, we evaluated 100 YouTube videos related to heart failure. Based on our evaluation tools, most YouTube videos on heart failure were found to be of very low quality, as reflected by the median DISCERN score. This highlights a clear need for improved educational content that adheres to evidence-based guidelines and is accessible to patients. Although the overall quality of the videos was low, the quality of videos uploaded by healthcare professionals was found to be higher across all three scoring systems (GQS, DISCERN, and JAMA). As the video duration increases, the quality of the video also improves, likely due to the opportunity to include more content. Video popularity does not have an effect on video quality. Considering the increasing access to and use of YouTube videos for information, videos uploaded to the platform should be carefully monitored.

## Data Availability

The datasets generated and/or analyzed during the current study are available from the corresponding author upon reasonable request.
